# Evaluation of the therapeutic effect of mesoporous silica nanoparticles loaded with Gallic acid on reserpine-induced depression in Wistar rats

**DOI:** 10.1186/s40360-022-00579-1

**Published:** 2022-06-15

**Authors:** Heba M. Fahmy, Eman R. Mohamed, Aida A. Hussein, Yasser A. Khadrawy, Nawal A. Ahmed

**Affiliations:** 1 Biophysics Department, Faculty of Science, Cairo University, Giza, Egypt; 2 Zoology Department, Faculty of Science, Cairo University, Giza, Egypt; 3 Zoology Department, Faculty of Science, Suez University, Suez, Egypt; 4 Medical Physiology Department, Medical Division, National Research Centre, Cairo, Egypt

**Keywords:** Gallic acid, Mesoporous nanoparticles, Depression, Monoamines, Oxidative stress, Rat

## Abstract

**Background:**

The current study evaluates the free gallic acid (GA) and GA-loaded mesoporous silica nanoparticles (MSNs) antidepressant efficacy in a rat model of depression caused by reserpine.

**Methods:**

By using a scanning electron microscope (SEM), dynamic light scattering (DLS), and zeta potential, MSNs and GA-loaded MSNs were characterized. The efficiency of encapsulation and the release of GA-loaded MSNs were also investigated. The effect of GA, either in its free form or loaded on (MSNs) on oxidative stress biomarkers and monoamine neurotransmitters levels (serotonin (5-HT), norepinephrine (NEP), and dopamine (DA)), were evaluated in these areas (cortex, hippocampus, striatum, and hypothalamus) of control, a depression model of rat, a depression model of rat treated with either free GA, MSNs or GA loaded MSNs. The forced swimming test (FST) also the open field test (OFT) were carried out to evaluate the behavioral changes in all groups.

**Results:**

Reserpine caused a decrease in the time spent in motor and swimming activity besides increasing the time of immobility, as demonstrated by OFT and FST. Significantly reductions in 5-HT, NEP, and DA were obtained in the cortex, hippocampus, hypothalamus, and striatum of reserpine-treated rats. Free GA was more effective in increasing the serotonin level in the cortex, hippocampus, and hypothalamus, while GA-loaded MSNs were more effective in increasing it in the striatum. GA-loaded MSNs also increased the level of NEP in the four studied brain areas. Free GA increased dopamine levels in the cortex and striatum, whereas GA-loaded MSNs increased DA levels in the hippocampus and hypothalamus compared with the depressed untreated group.

**Conclusions:**

MSNs can be used as a drug delivery system to target GA selectively to specific brain areas.

## Background

Depression can be defined as a mood disorder accompanied by unpleasant, helpless, sad, and desperate feelings [1]. Depression is regarded as a severe medical condition, and without adequate therapy, it can worsen. A deficiency in monoamine neurotransmitters (5-HT, NEP, and DA) is one of the main causative factors for depression. Most antidepressants alleviate depression by increasing the levels of these neurotransmitters, which are affected in slightly distinct ways by each class of antidepressants [2]. Several specific antidepressant drugs are available that similarly exhibit effectiveness, although there is a continuous discussion about their efficacy compared with a placebo [3–5]. As a result, choosing an antidepressant primarily depends on considering future negative consequences. Current guidelines recommend strict serotonin reuptake inhibitors as first-line depression therapy [6].

According to reports, one of the fundamental processes involved in developing depression is oxidative stress [7]. Excessive reactive oxygen species (ROS) buildup and insufficient antioxidant enzymes can cause significant cellular damage. As a result, illnesses such as depressive disorders may develop [8, 9]. Many synthetic antioxidants are widely prescribed, but they must be used under strict legislation because of their potential risks in vitro. Naturally occurring antioxidants that inhibit toxicants have garnered significant attention [10]. Many health professionals prefer natural antioxidants, and patients are looking for additional antioxidants, mainly plant extracts, such as green tea, rosemary, and olive oil [11].

Gallic (GA) and its esters are components of a common plant characterized by their antioxidant activity (3,4,5-tri hydroxybenzoic acid GA). The interfacial radical chemistry of the GA’s phenolic group leads to GA’s antioxidant activity [12]. GA is one of the main polyphenolic substances in crops, berries, mangos, walnuts, green tea, and wine [13]. GA exerts anti-anxiety [14], antidepressant [15], and anti-epileptic [16, 17] effects. Neuroprotective and antioxidant effects of GA acid derivatives have also been documented [18]. GA is known as a powerful natural antioxidant that scavenges reactive oxygen species (ROS) (e.g., anions of superoxide, peroxide (H_2_O_2_), radicals containing the hydroxyl group, and hydrochloric acid) [19]. Antioxidant instability and solubility are important issues in pharmacology. Therefore, choosing an appropriate carrier for an antioxidant is necessary to make it more stable and keep it from interacting with other molecules in the bloodstream [20].

Unlike typical formulations, which result in instant and spontaneous drug release and require frequent administration, medication delivery using nanoparticles may be adjusted and maintained to increase target accessibility. Functionalization can improve these characteristics by employing cleavable agents or using “gatekeepers” for capping porous particles. Also, active targeting may be achieved by attaching nanoparticles to antibodies or ligands, thereby reducing non-specific drug toxicity by indirectly decreasing the levels in tissues that are not targeted [21].

Silica nanoparticles are common inorganic drug carriers because of their increased surface absorption, enhanced surface activity, and low demand for sol-gel technology [22]. Mesoporous silica nanoparticles (MSNs) are inorganic nanoparticles with many advantages for drug delivery [23–25]. Pore diameters controlled in MSNs ranging from 2 to 50 nm have a well-defined pore setting [21]. Pores can be created to accept medicinal ingredients imagery or two and can be employed for a considerable fraction of the total capacity. The former may increase immunization with drugs, whereas the latter helps improve suspension balance and induce regulated and continuous release (i.e., capping pore openings) [26].

For various reasons, using MSNs for medication administration and medical imaging applications may be more advantageous than using other types of nanoparticles. Silica and silicon compounds used to make MSNs, come in various forms and are considered harmless, biocompatible, and biodegradable [27]. MSNs have easy-to-control pore size and structure, surface functionalization, and morphology [28]. MSNs have superior stability in terms of degradation and resilience to external stress than other nanoparticles, such as dendrimers, liposomes, and niosomes, due to their stable SiO bonding [29]. MSNs have a high drug loading capacity, transport loaded medications to target tissues without leaking them, improve the bioavailability of poorly soluble pharmaceuticals, and protect loaded drugs from gastrointestinal fluids and acids [30].

Plant gallic acid (GA) is a fascinating medicinal component in previous work. GA’s bioavailability and residence time in the body system is greatly reduced due to its short lifespan and autoxidation in an aqueous solution. GA was chemically bonded to silica nanoparticles in this study, and a silica nanoparticle drug delivery system was designed to control GA release based on the hydrolysis of the chemical bonds. As a result of this research, a unique drug delivery method for GA with controlled release and extended antioxidant efficacy was developed [31].

Pharmaceutical researchers face substantial issues with antioxidant stability and solubility in another work. As a result, utilizing a good carrier for an antioxidant can improve antioxidant stability while also protecting it from reacting with other molecules in the bloodstream. Because of their favorable biological features, mesoporous silica nanoparticles (MSNs) have been widely exploited as a carrier for therapeutic applications. Different amounts of GA (1–50 mg/mL) in EtOH were produced, taking samples after 24 and 48 hours. According to the results, the best GA loading capacity was found at a concentration of 40 mg/mL in 48 hours. Spectrophotometry and high-performance liquid chromatography (HPLC) examination revealed that the maximal GA loading capacity and entrapment efficiency were 46 and 20%, respectively [20].

This study investigated the fundamental mechanics to see if GA-loaded MSNs had any antidepressant properties in a Wistar adult male rat depression model produced by reserpine exposure.

## Materials and methods

### Chemicals

CTAB (N- cetyl trimethyl ammonium bromide), TEOS (Tetra ethoxy orthosilicate), DMSO (Dimethyl sulfoxide), Thiobarbituric (TBA) acid, and reduced glutathione were obtained from Sigma Aldrich, Germany. 1-Chloro-2,4- Dinitrobenzene (CDNB), acetyl thiocholine iodide (CH_3_COSCH_2_CH_2_N(CH_3_)_3_I) (about 99%), o-phthaldehyde, Heptane, and Acetic acid were obtained from Sigma Aldrich, St. Louis, USA. SDFCL (SD Fine-Chem Limited) in Egypt provided the trichloroacetic acid (TCA). Bio Diagnostic Co., Giza, Egypt, provided the following samples, (100 mM / l) phosphate saline buffer (PSB) pH at 7.4, (phenazine methosulfate (PMS), Sodium pyrophosphate, Nitro blue tetrazolium, and NADH (Nicotinamide adenine dinucleotide reduced) were obtained from Bio Diagnostic Co., Giza, Egypt.70% Perchloric Acid, Sulfanilamide (C_6_H_8_N_2_O_2_S), were bought from Central Drug House (CDH), India. Disodium Edetate (Na_2_EDTA), Sodium Dihydrogen Phosphate (Na. H_2_PO_4_) (98%), and Disodium Phosphate (Na_2_HPO_4_) were purchased from EL Nasr Pharmaceutical Chemicals Co (ADWIC), Egypt. Sodium hydroxide (NaOH) was bought from chemicals firm Alamia, Egypt. N-1-naphthylene diamine dihydrochloride, 85% of Phosphoric Acid, and 99% of dipotassium Hydrogen Phosphate (K_2_HPO_4_) were bought from El-Gomhoria Co For Pharmaceutical, Egypt. 5,5′ dithiobis-(2-nitrobenzoic acid) (DTNB) (99%) was bought from Acros Organics, USA. Potassium dihydrogen (KH_2_PO_4_) (99%) was bought from NICE chemicals, India. 96% Ethyl Alcohol (Ethanol) was purchased from Diachem Chemicals, USA.

### Methods of preparation

Mesoporous silica nanoparticles were made with the edited Stober method; firstly, in 350 mL of clean water, N- CetylTri methyl ammonium bromide (2.5 g of CTAB) was dissolved. Then, 5 mL of NaOH (28%) was applied to the CTAB solution at room temperature. The mixture was heated at 80 °C for half an hour, then at this temperature (80 °C), 12.5 ml of TEOS was dropped into the CTAB solution at a rate of 1 ml/min. The completed mixture was rapidly agitated at room temperature for 24 hours before being refrigerated. A white precipitate was obtained and separated by centrifugation, then rinsed with plenty of water and ethanol and dried in a vacuum oven. The surfactant was eliminated by calcining at 540oC for 6 hours at a 1 °C/min heating rate [32].

### Loading of Gallic acid on the prepared MSNs

For loading GA on the prepared MSNs, GA in ethanol (40 mg/ml) was prepared, and in a vial containing 1 ml GA in ethanol, 10 mg of MSNs were administered. At room temperature, the vial was placed on the shaker at 155 rpm for 48 hours in the dark. After that, MSNs loaded with GA were centrifuged, rinsed three times with ethanol, then vacuum-dried at room temperature. This supernatant was gathered for GA loading determination [20]. The loading capacity was calculated as follows:

$$\text{Loading capacity}\;\hspace{0.5em}\%=\frac{\left(\text{Initial concentration}-\text{Final concentration}\right)}{\left(\textit{Nanoparticles weight}\right)\times100\hspace{0.5em}\%}$$

The gallic acid (25 mg) was dissolved in 1 ml of DMSO: saline solution at a 1:9 ratio. The produced GA was added in a 1:1 weight ratio to the MSNs dissolved in deionized water. For 24 hours, this suspension was shaken at 100 rpm in a water bath at room temperature. GA-loaded MSNs were then isolated using centrifugation. A calibration curve was used to calculate the concentration of free GA in the supernatant at a wavelength of 272 nm [33].

### MSNs and GA-loaded MSNs characterization

The encapsulation efficiency (EE%) of GA was calculated for GA-loaded MSNs. Centrifugation was used to separate the GA-loaded MSNs from the ethanol solution, followed by three ethanol washes and drying under vacuum at room temperature. The supernatant was collected. Free GA in the supernatant was dissolved in ethanol, and After that, a UV visible spectrophotometer was used to detect the absorbance at 272 nm (Jenway 6405, U.K.). The HPLC study also validated UV-Vis spectroscopy findings [34]. The encapsulation efficiency was calculated for GA-loaded MSNs from the following equation:

$$\textit{Encapsulation Efficiency}\;\hspace{0.5em}\%=\frac{\left(\textit{Initial concentration}-\textit{Final concentration}\right)}{\left(\textit{Initial concentration}\right)\times100\%}$$

### In vitro release of GA

To study the release of GA from GA-loaded MSNs, Inside a vial containing aqueous PBS solutions (4 ml) and ethanol (1 ml), 10 mg of GA-loaded MSNs were prepared. The prepared vial containing GA-loaded MSNs was put in 15 ml of PBS and stirred at 80 rpm for 48 hours at 37 °C, with the 2 ml of dissolving medium being replaced with PBS every 30 minutes at pH 7.4 till the whole release period was finished. Sampling was done at predetermined periods, and the dissolution medium was instantly substituted with the new medium. In a UV– vis spectrometer (Jenway UV-6420; Barloworld scientific, Essex, UK) at 274 nm, the quantity of releasing GA in the supernatant was examined [35].

### Physical characterization of the prepared formulations

#### Scanning Electron Microscopy (SEM)

Particle structural characterization was determined using a scanning electron microscope (Philips XL-30) [34].

#### Particle size and zeta potential

The size of the nanoparticles and zeta potential of the produced MSNs and GA-loaded MSNs formulations were measured using the dynamic light scattering (DLS) analysis (Zetasizer Nano ZS90, Malvern Instruments, UK) at 25 °C. After the suitable dilution with deionized water, the particle size of MSNs and GA-loaded MSN was evaluated. The hydrodynamic diameter and total surface charge (Z-potential) of MSNs and GA-loaded MSNs were determined using the dynamic light approach by suspending them in ethanol at a 1 mg/ml [22]. The zeta-potential was measured using the same capillary cuvette directly after the particle size measurement. The refractive index has been held at 1.33, and the viscosity at 0.89 cp has been maintained to imitate the pure water values. The assessments were carried out in triplicate in the sample, and the results were then represented as the mean ± S.E.M.

#### In vivo studies

##### Animals

In the current work, 30 Wistar rats, mature males weighing 150–200 g, were used. They were lodged under standardized temperature and humidity conditions in individual cages. The institutional Animal Ethics Committee approved all in vivo experimental procedures (Registration no.: CUIF 7318).

##### Experimental design

The rats in this experiment were separated into five groups consisting of 6 rats. Group I: negative control group (normal rats) that got intraperitoneal injections daily (i.p) of the standard solution of saline (0.9%) during the whole experimental time. Group II: depressed rats caused by a daily reserpine i.p. injection (0.2 mg/kg) during the first 14 days, then every two successive days until the end of the experimental time (for another10 days). Group III: depressed rats treated daily with reserpine for 14 days followed by i.p. injections of free GA daily (20 mg/kg) during the 10 days. Group IV: depressed rats treated daily with reserpine for 14 days followed by i.p. injections of free MSNs daily (20 mg/kg) during the 10 days. Group V: depressed rats treated daily with reserpine for 14 days followed by i.p. injections of GA-loaded MSNs daily (20 mg/kg) during the 10 days. During the treatment period (10 days) in groups III, VI, and V, rats got a 0.2 mg/kg of reserpine every two successive days. Free MSN and GA-loaded MSNs solutions were prepared by dissolving MSN or GA-loaded MSNs in a few drops of DMSO, and the solutions were completed with saline.

A forced swimming test and an open-field test were used to assess each rat’s behavior when the experiment was finished. Then, rats were decapitated, and the cortex, hippocampus, hypothalamus, and striatum were dissected, weighed, and stored at − 20 C° until neurochemical tests were performed.

#### Behavioral assessment

##### Forced Swimming Test (FST)

During the FST experiment, Separate swimming sessions were needed for rats in a cylinder (height is 50 cm and diameters are 22.5 cm) with open Plexiglas at room temperature (23–25 °C), and it was filled with water to the tune of 75% of its total volume so the rodents could not escape. Each rodent was gently and gradually immersed in the water. The test period was 5 min for each rat. The floating, swimming, and struggling time for each rat was recorded. Before the test, the rats learned to swim in a training session. This experiment was performed on the 13th induction day and again on the 10th day after treatment [36].

##### Open Field test (OF)

The test area was 66 × 66 cm^2^ with 30-cm high surrounding walls. There were 36 squares in the OF arena. The region in the center accounted for 25% of the arena’s general area. The time limit for the exam was set at 10 min. The number of crossed squares and time spent in the central region, rearing, and grooming were recorded [36].

#### Neurochemical measurements

Each part of the brain was homogenized. In cold potassium phosphate buffer, its pH is 7.4 to yield a 10% uniform suspension to measure malondialdehyde (MDA), nitric oxide (NO), and reduced glutathione levels (GSH), also the activities of catalase (CAT), superoxide dismutase (SOD), glutathione-S-transferase (GST), and acetylcholine esterase (AChE).

#### Determination of the activity of superoxide dismutase

Superoxide dismutase (SOD) activity was measured using a purely chemical procedure. This examination is dependent on the enzyme’s competence to inhibit the degradation of nitro-blue tetrazolium dye induced by phenazine methosulfate. The absorbance shift was assessed over 5 min at 560 nm [37].

#### Measurement of reduced glutathione (GSH) levels

This method involves reducing Ellman’s reagent [38] to produce 2-nitro-s-mercaptobenzoic acid, a yellow color resulting from the SH groups in GSH. The absorbance was measured at 412 nm (Jen way UV- 6420; Barlo world scientist, Essex, UK), and the GSH levels were determined relative to a calibration curve with values ranging from 1 to 6 mmol.

#### Detection of glutathione-s-transferase activity (GST)

The glutathione-S-transferase (GST) activity was measured using an assay of The Habig (1974). In a 37 °C water bath, 0.4 ml of 50 nM sodium phosphate buffer (pH 6.5) was combined with 1.2 ml water, 0.1 ml supernatant, and 0.1 ml of 30 mM 1-chloro-2,4-dinitrobenzene (CDNB). After incubation, 0.1 ml of 30 mM reduced glutathione was added. At 340 nm, a change in absorbance was measured [39].

#### Measurement of nitric oxide levels

Using the method of Moshage et al., Griess reagent was used to determine (NO) nitric oxide levels by measuring nitrite [40]. The supernatant was mixed with Griess reagent, and the nitrite was converted into an azo compound with a rich violet hue. The absorbance at 450 nm was calculated using a spectrophotometer type (Jen way UV- 6420; Barlo world medical, Essex, UK). This assay uses the stable NO radical product, nitrite, as a NO indicator.

#### Determination of malondialdehyde (MDA) levels

MDA levels were calculated according to Ruiz-Larrea et al. [41]. The reactive components of thiobarbituric acid were tested as a lipid peroxidation indicator. Thiobarbituric acid reacts with reactive compounds in this assay to generate a pink compound measured at 532 nm using a spectrophotometer.

#### Determination of acetylcholine esterase (AChE) activity

A modified method by Ellman et al. [42] was used to measure AChE activity represented by Gorun et al. [43]. The test detects thiocholine, which is a hydrolyzed acetylthiocholine derivative. The reagent, 5,5′-dithiobis(2-nitrobenzoic acid) (DTNB), interacts with thiocholine and is converted to a reduced version (5-thio-2-nitrobenzoic acid) to form a yellow anion at a wavelength of 412 nm.

#### Measurement of monoamines levels

The cortex, hippocampus, hypothalamus, and striatum were homogenized in a freezing environment with acidified n-butanol solution and centrifugation for 5 minutes at 2000 rpm. 2.5 mL supernatant was combined with 1.6 mL acetic acid (0.2 N) and 5 ml of heptane. The tubes were vortexed lasting 30 seconds, and centrifugation for 5 minutes at 2000 rpm. The organic layer was eliminated, and 200 μl of the liquid layer was transported to vials to measure serotonin using o-phthalaldehyde. Also, 1 ml of the liquid phase was transferred to vials to measure NEP and DA using a fluorometric method [44]. A spectrofluorometer (Jasco – FP 6500 model, Japan) with a 150 W xenon arc lamp as the source was used (the excitation slit bandwidth was 5 nm, and the emission slit bandwidth was 5 nm).

### Statistical analysis

The OF, forced swimming, monoamines, and oxidative stress marker results are provided as the standard error of the mean (mean ± S.E.M) and examined by a comparative analysis. The F-test and Duncan’s post hoc were also performed, and SPSS software was used to examine the data (available in version 16.0). *P*-values < 0.05 were determined to be significant statistically.

## Results

### Characterization of the physicochemical properties of the formulations

#### Drug encapsulation efficacy and in vitro release of GA

**Fig. 1:**
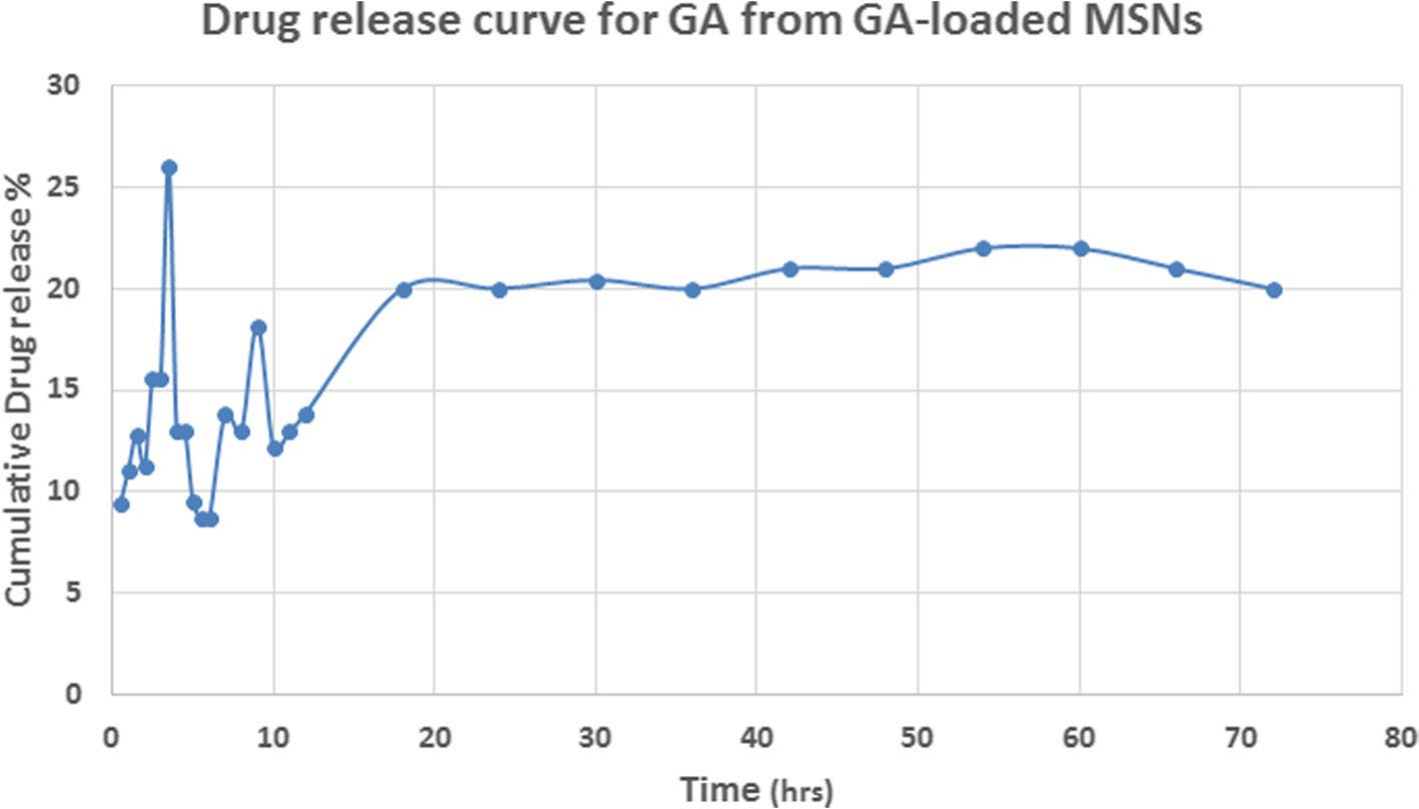
Drug release curve of GA-loaded MSNs

GA released from the GA-loaded MSNs in vitro was evaluated to gain insight into its pharmacokinetic properties. The efficacy of GA loading was 83.86% for GA-loaded MSNs, indicating high drug encapsulation in the nanoparticles. Approximately 26% of GA was released from the MSNs within 4 hrs, and GA was released rather quickly and continuously for the next 72 h (Fig. 1).

#### Particle size

**Fig. 2:**
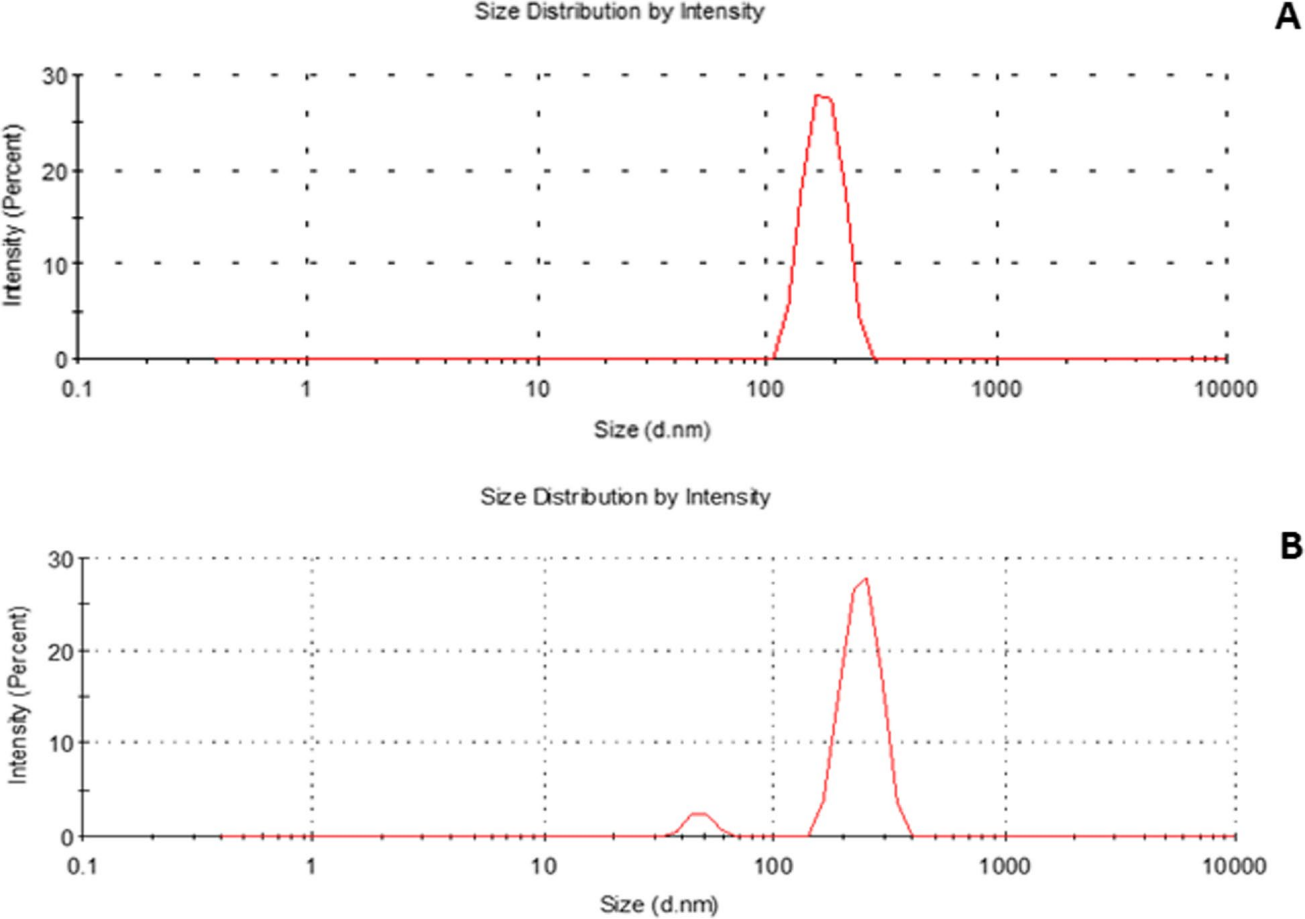
Particle size distribution of: (**A**) free MSNs and (**B**) GA-loaded MSNs using dynamic light scattering technique

Figure 2A and B show that the median hydrodynamic diameters for the free and GA-loaded MSNs were 178.3 ± 1.4 and 241.6 ± 42.10, respectively. The index of polydispersion (PDI) measures particle size uniformity and homogeneity. The polydispersity index (PDI) distribution was 0.447 for free GA and 0.48 for GA-loaded MSNs.

### Zeta potential

**Fig. 3:**
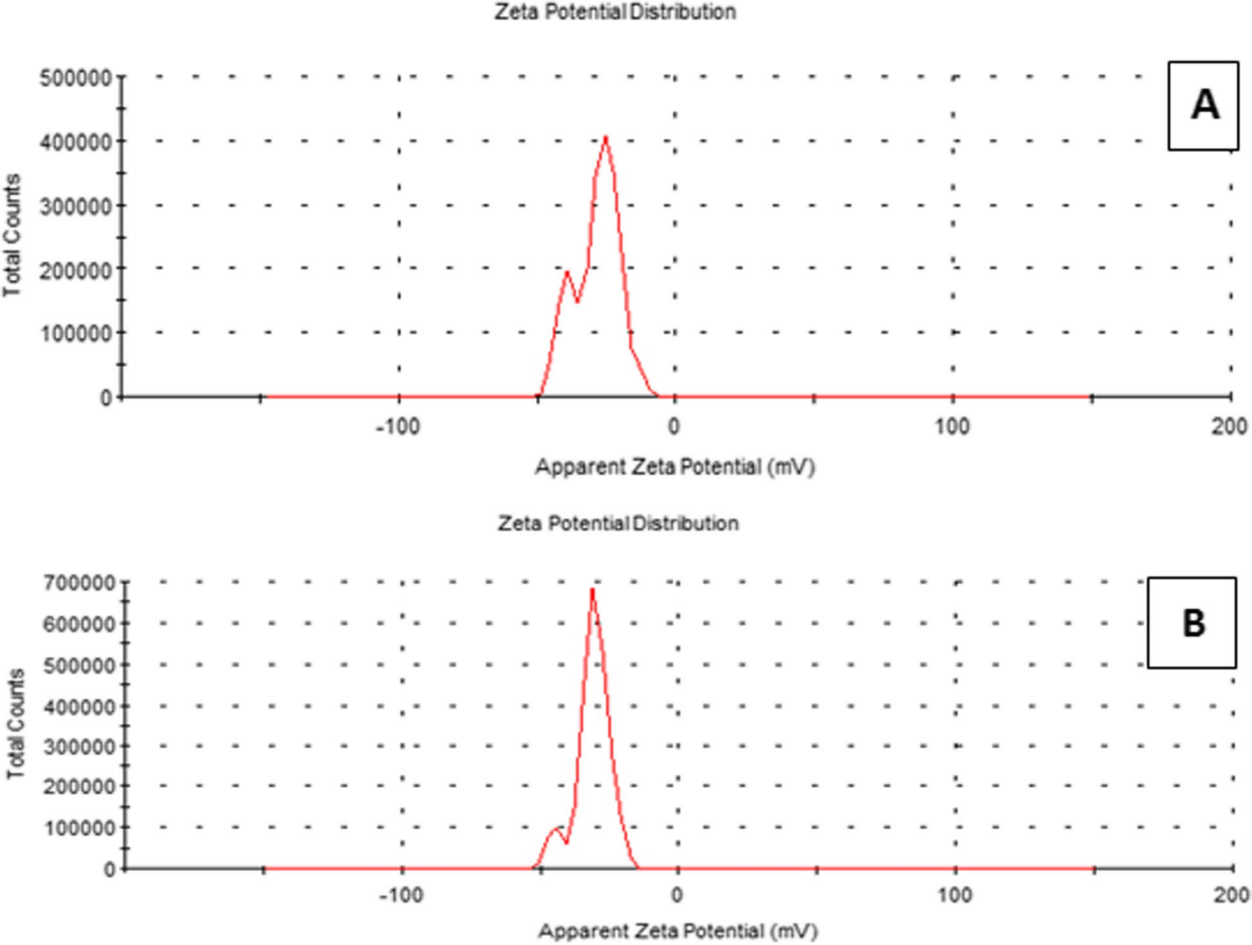
Zeta potential distribution of: (**A**) free MSNs and (**B**) GA-loaded MSNs using dynamic light scattering technique

The zeta potential scale measures how far repulsive forces between neighboring, identically charged particles disperse. The mean zeta potential of the MSN was − 29.9 ± 3.23 mV, whereas that for GA-loaded MSNs was − 31.4 ± 2.8 mV, as shown in Fig. 3A and B.

### Scanning Electron Microscope (SEM) imaging

**Fig. 4:**
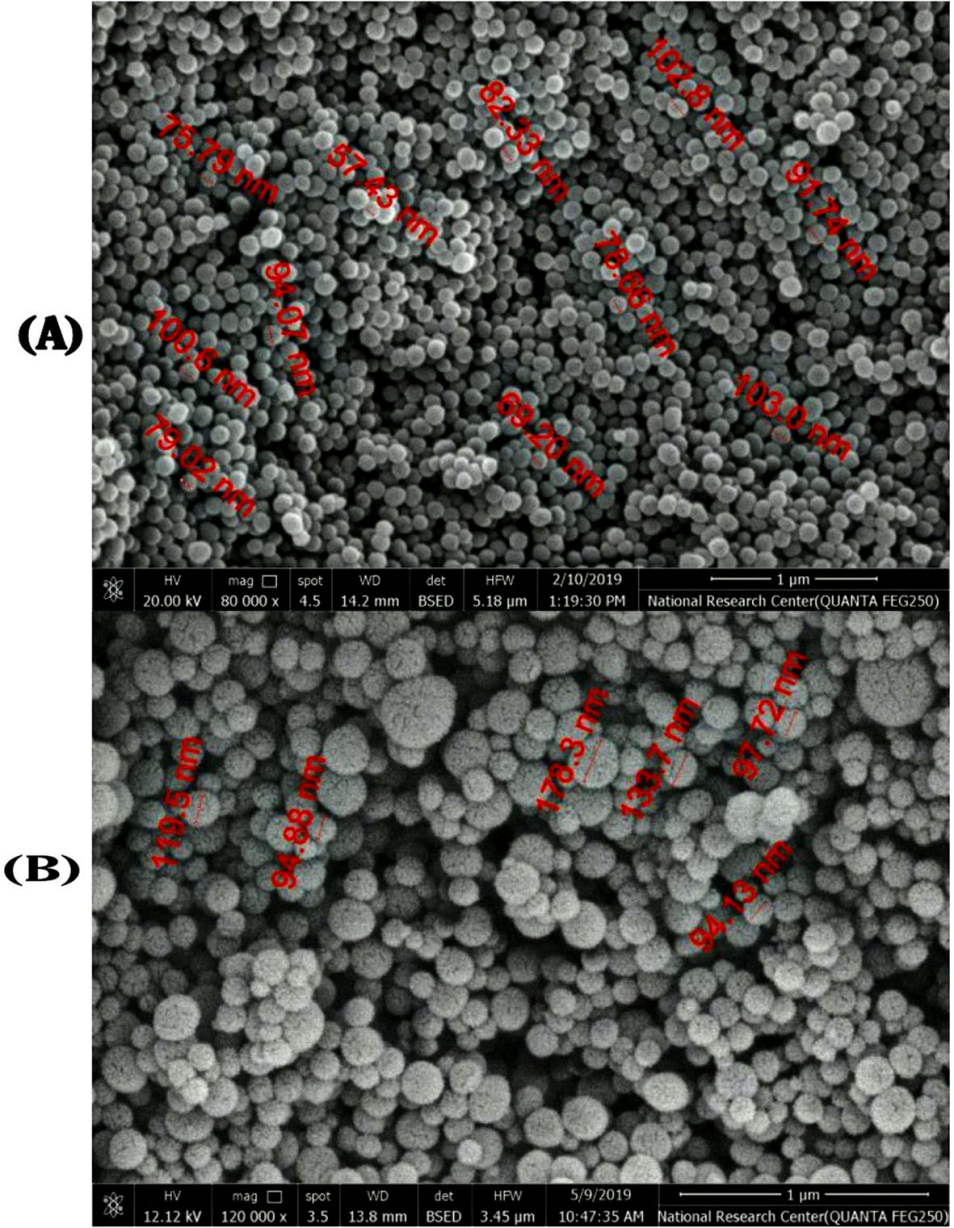
Scanning electron micrograph (SEM) of: (**A** and **B**) free MSNs and (C and D) GA-loaded MSNs

As determined by SEM, the MSNs and GA-loaded MSNs’ approximate size was 84.96 ± 4.46 and 119.7 ± 13.4 nm, as shown in Fig. 4A and B. The MSNs were within the nanometer scale. The morphology of the MSNs appeared as a smooth surface with few spherical aggregations.

### Behavior tests

FST and OFT were used to evaluate the behavioral effects of antidepressant activity.

#### The OF test

**Fig. 5:**
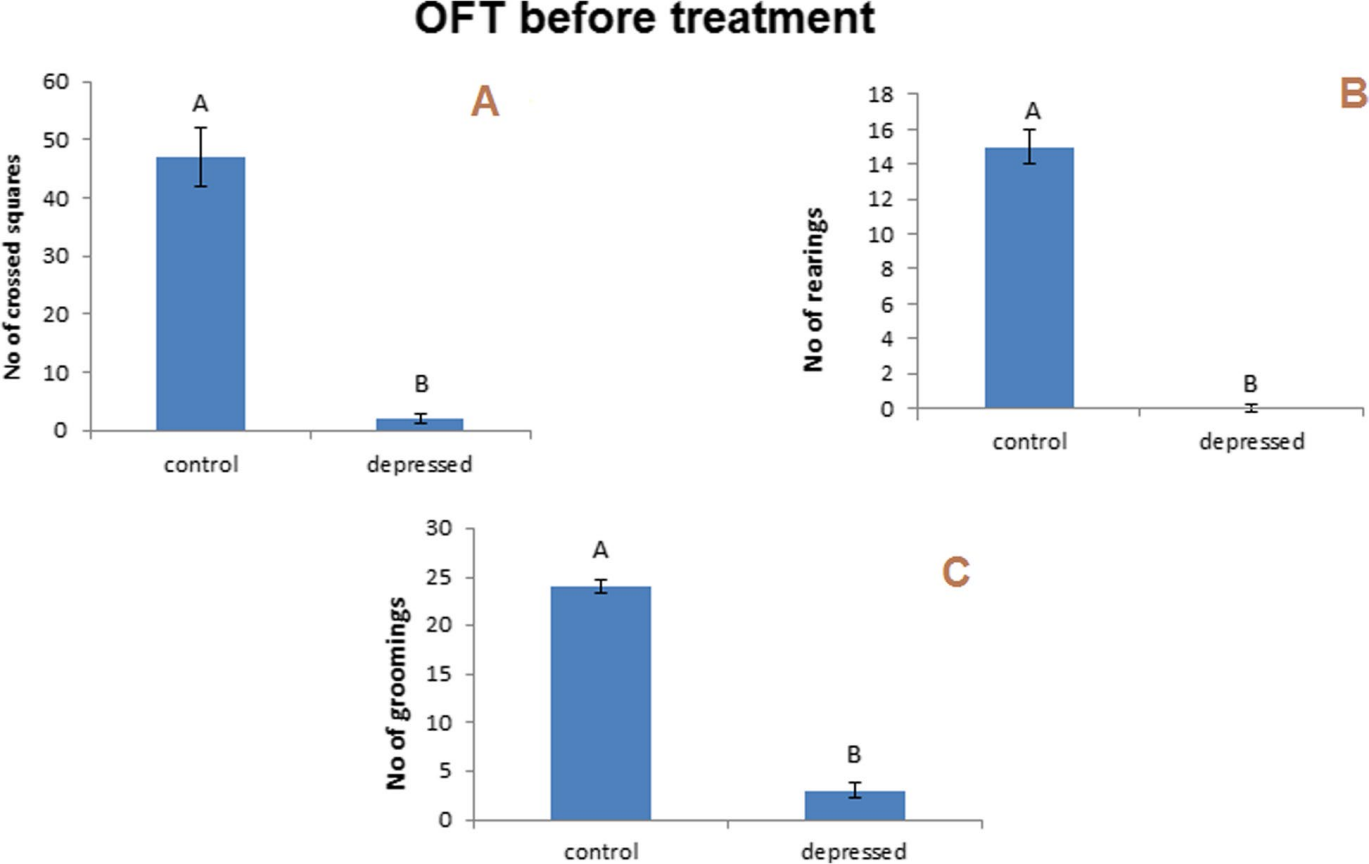
Effect of i.p. injection of reserpine on the behavior in open field test before treatment

**Fig. 6:**
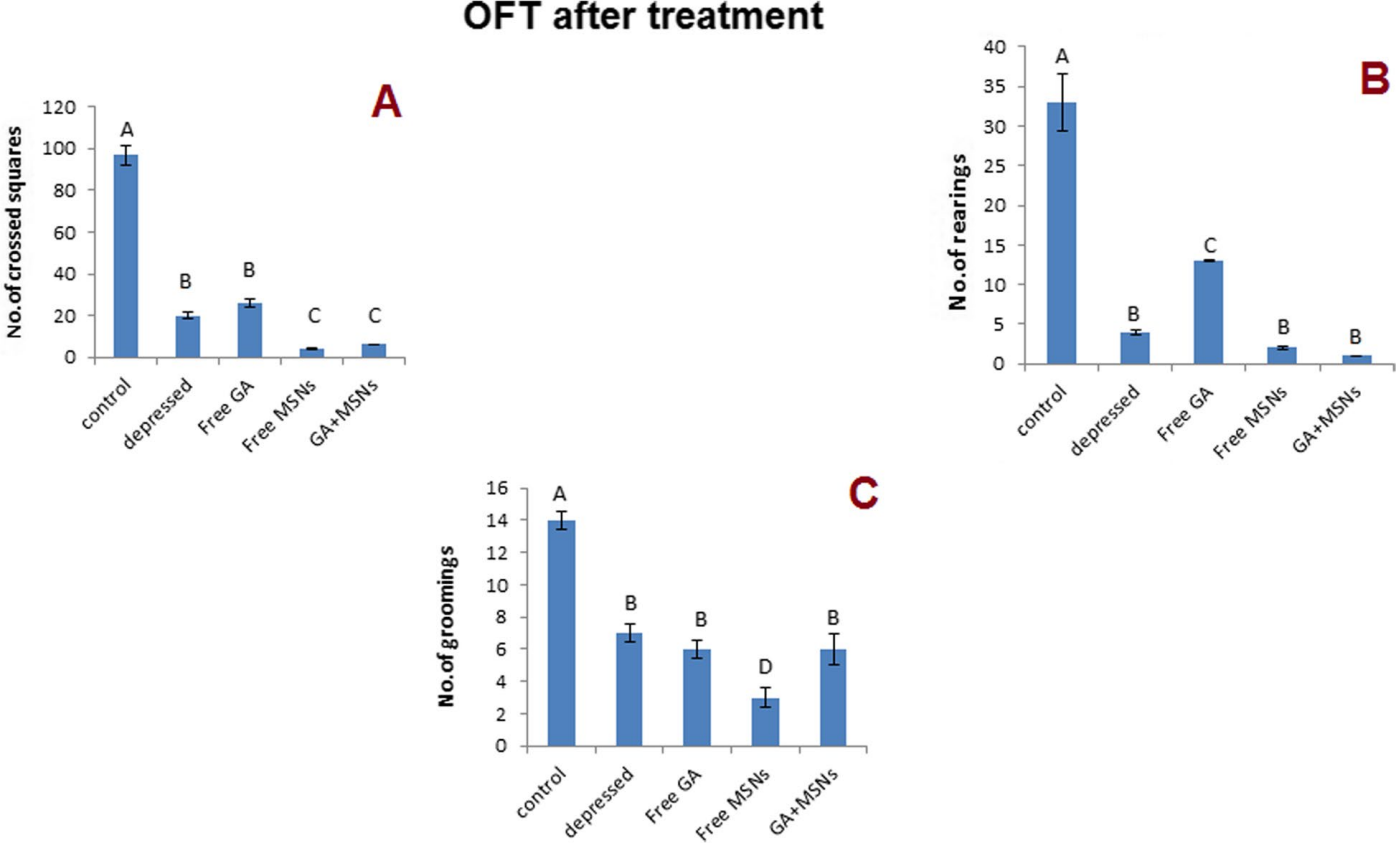
Effect of i.p. injection of reserpine, free GA, free MSNps, GA-loaded MSNPs on the behavior in open field test after treatment

The decreasing number of squares crossed, rearing, and grooming is considered the marker for depression. This study showed a considerable reduction in crossing squares, rearing, and grooming (*P* < 0.05) after 14 days of reserpine therapy daily. The levels were decreased significantly compared with the control rats (− 96, − 100, and − 88%, respectively) (Fig. 5). After 10 days of daily treatment with different MSN formulations, all treated groups exhibited significant decreases in the number of squares crossed relative to the control group (Fig. 6A). All treated groups exhibited significant decreases in rearing compared to the control group (Fig. 6B). Of note, all the treated groups showed significant decreases in grooming compared to the control group (Fig. 6C).

#### FST

**Fig. 7:**
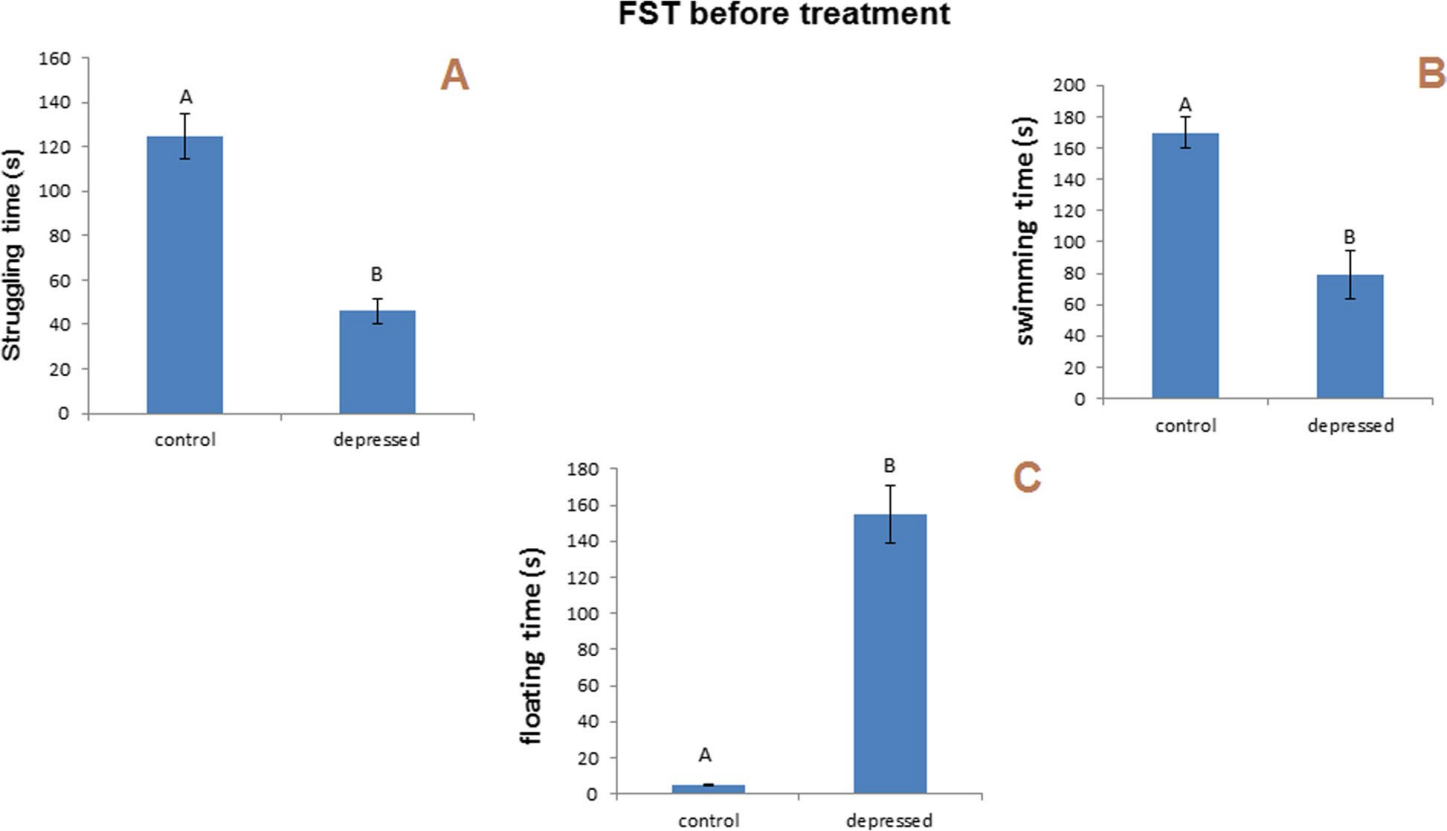
Effect of i.p. injection of reserpine on the behavior in the forced swimming test before treatment

**Fig. 8:**
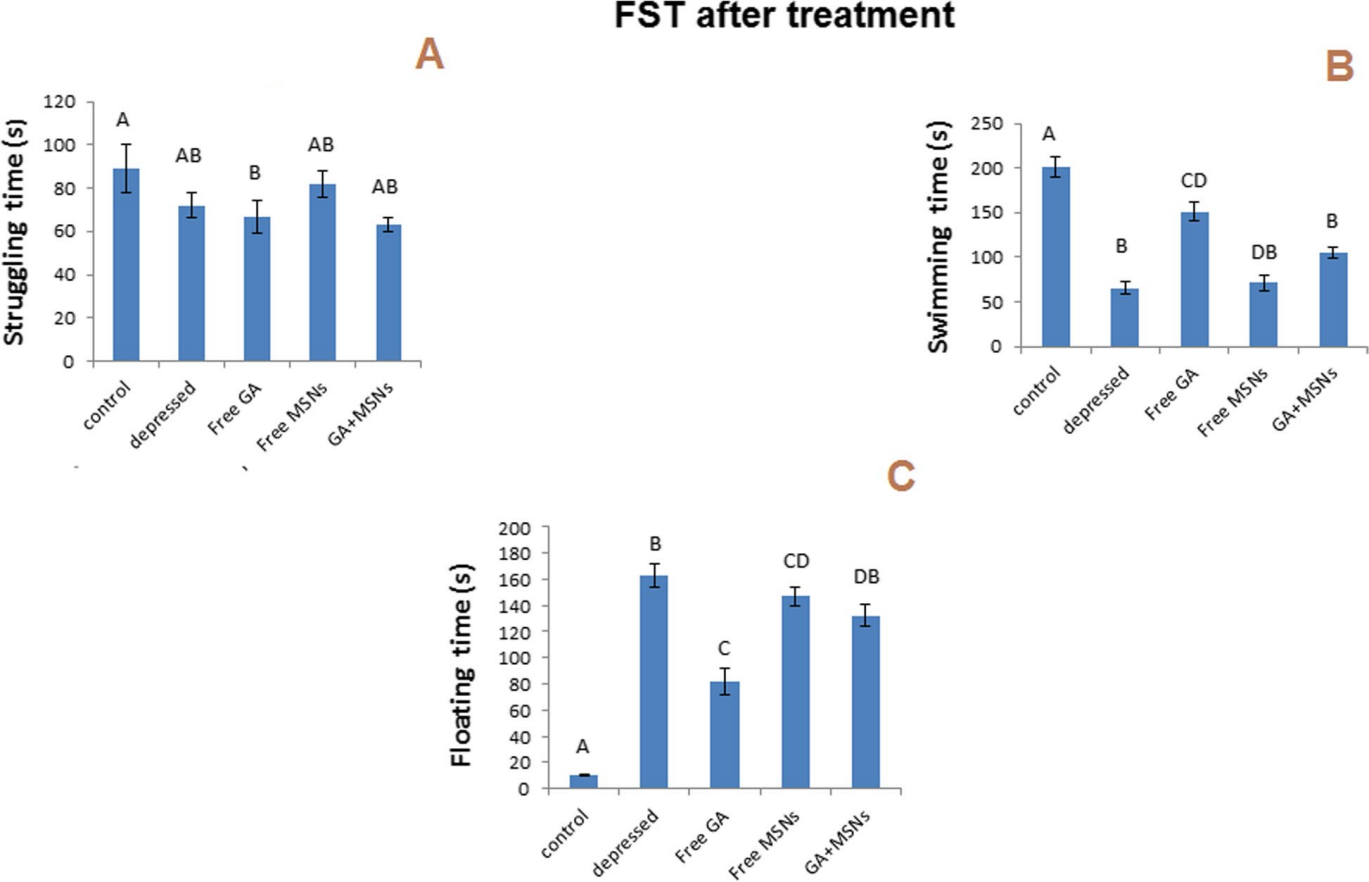
Effect of i.p. injection of reserpine, free GA, free MSNps, GA-loaded MSNPs on the behavior in the forced swimming test after treatment

Raising in the floating time and reducing swimming and struggling time is considered an indicator of depression. In the following research, for the swimming test, the results interested a significantly reduced (P less than 0.05) duration of struggling and swimming (− 63, − 54%) time and a significantly increased time spent floating (3000%) after 14 days of daily reserpine administration compared with the controls (Fig. 7). Swimming time was significantly reduced for the three treated groups compared to the control group (Fig. 8B). The floating time increased significantly for the three treated groups compared to the control group (Fig. 8C). After 10 days of daily treatment with different formulations, insignificant decreases in struggling time were still observed for the group treated with free MSNs and GA-loaded MSNs, whereas struggling time was reduced significantly for the rats treated with free GA compared to a control group (Fig. 8A).

### Neurochemical study

#### Monoamine neurotransmitters

**Fig. 9:**
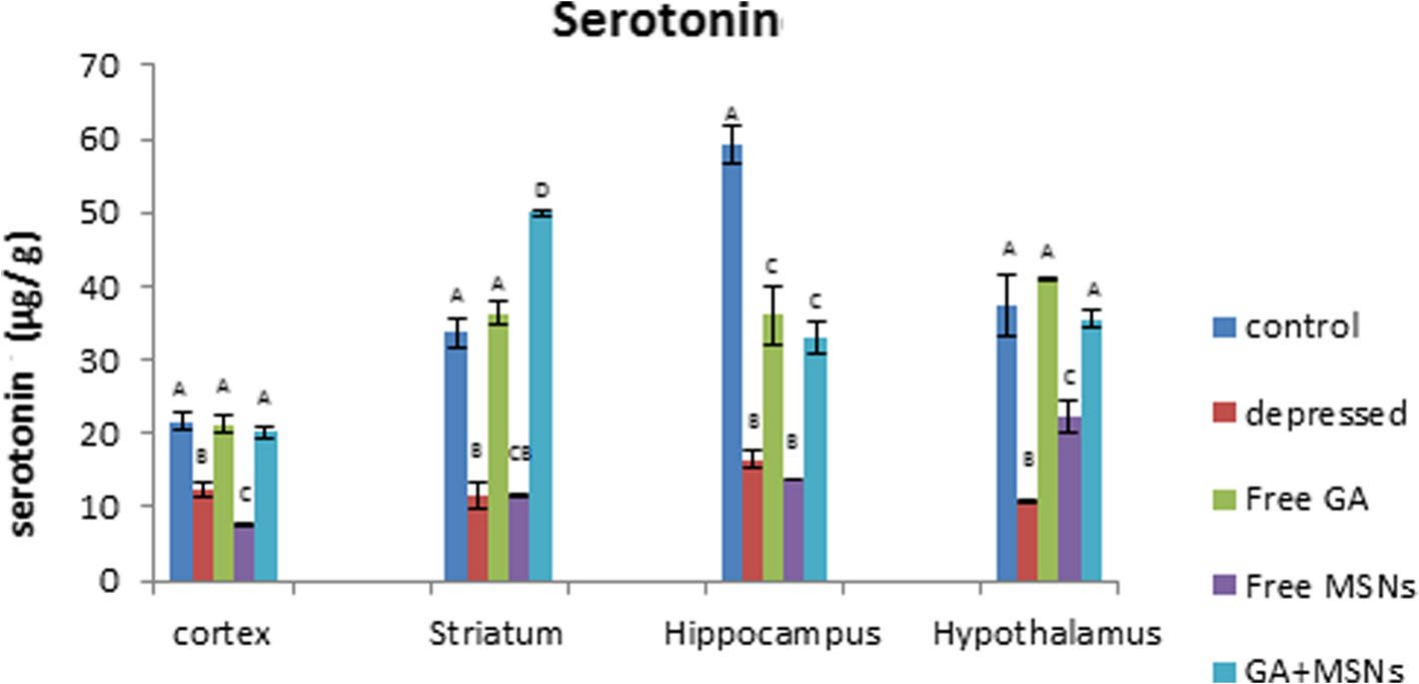
Effect of i.p. injection of reserpine, free GA, free MSNps, GA-loaded MSNPs on the serotonin levels in different brain areas of adult male Wistar rats

As shown in Fig. 9, using the reserpine-induced rat depression model, In comparison to the control group, a significantly reduced serotonin level was seen in the cortex (− 43%), striatum (− 66%), hippocampus (− 72%), and hypothalamus (− 71) regions. Treatment of depressed rats with free GA or GA-loaded MSNs restored serotonin levels to that of the controls. Decreased striatal serotonin levels were reversed to a significant increase using GA-loaded MSNs. Free GA and GA-loaded MSNs failed to restore serotonin levels to controls in the hippocampus. When comparing serotonin levels in the free GA- and GA-loaded MSNs-treated groups with the depressed untreated group, all brain areas exhibited increased serotonin levels. MSN treatment did not affect the reserpine-induced decreased serotonin levels in the cortex, hippocampus, striatum, or hypothalamus. Compared with untreated rats, MSNs resulted in a significant decrease in serotonin levels in the cortex, whereas insignificant changes were observed in the striatal and hippocampal regions. The hypothalamus was the only area that exhibited a significant increase in serotonin levels compared with untreated rats.

**Fig. 10:**
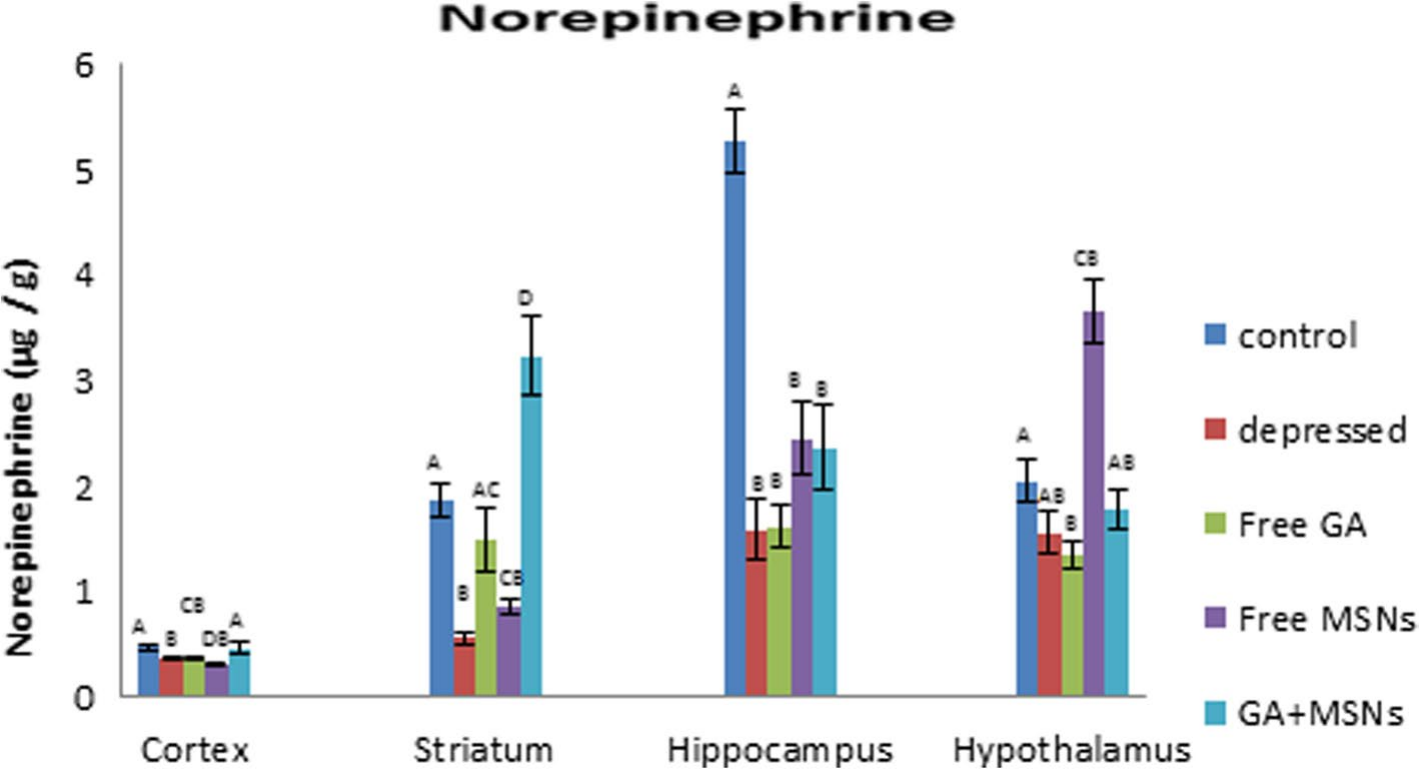
Effect of i.p. injection of reserpine, free GA, free MSNps, GA-loaded MSNPs on the NE levels in different brain areas of adult male Wistar rats

As shown in Fig. 10, reserpine decreased norepinephrine significantly in the cortex (− 23%), hippocampus (− 70%), and striatum (− 70%) regions, but not significantly in the hypothalamus (− 23%). Treatment with free GA did not restore NEP values in these areas; however, it restored striatal NEP to insignificant levels compared with the control. Compared with depressed rats, GA increased NEP non-significantly in the cortex, hippocampus, and striatum regions, whereas it was reduced insignificantly in the hypothalamus. In the depressed rats treated with free MSNs, NEP levels exhibited an insignificant reduction in the cortex, striatum, hippocampus, and a significant elevation in the hypothalamus compared to the controls. Compared with the untreated group, free MSNs reduced NEP levels insignificantly in the cortex, increased NEP insignificantly in the striatum and hippocampus, and significantly in the hypothalamus. GA-loaded MSN treatment restored NEP values in the cortex and hypothalamus to controls and increased striatal NEP significantly (72%) above the control value. GA-loaded MSN treatment failed to return NEP to control levels in the hippocampus. Compared with the depressed untreated group, GA-loaded MSN treatment exhibited significantly increased NEP values in the cortex and striatum areas and insignificant increases in the hippocampus and hypothalamus.

**Fig. 11:**
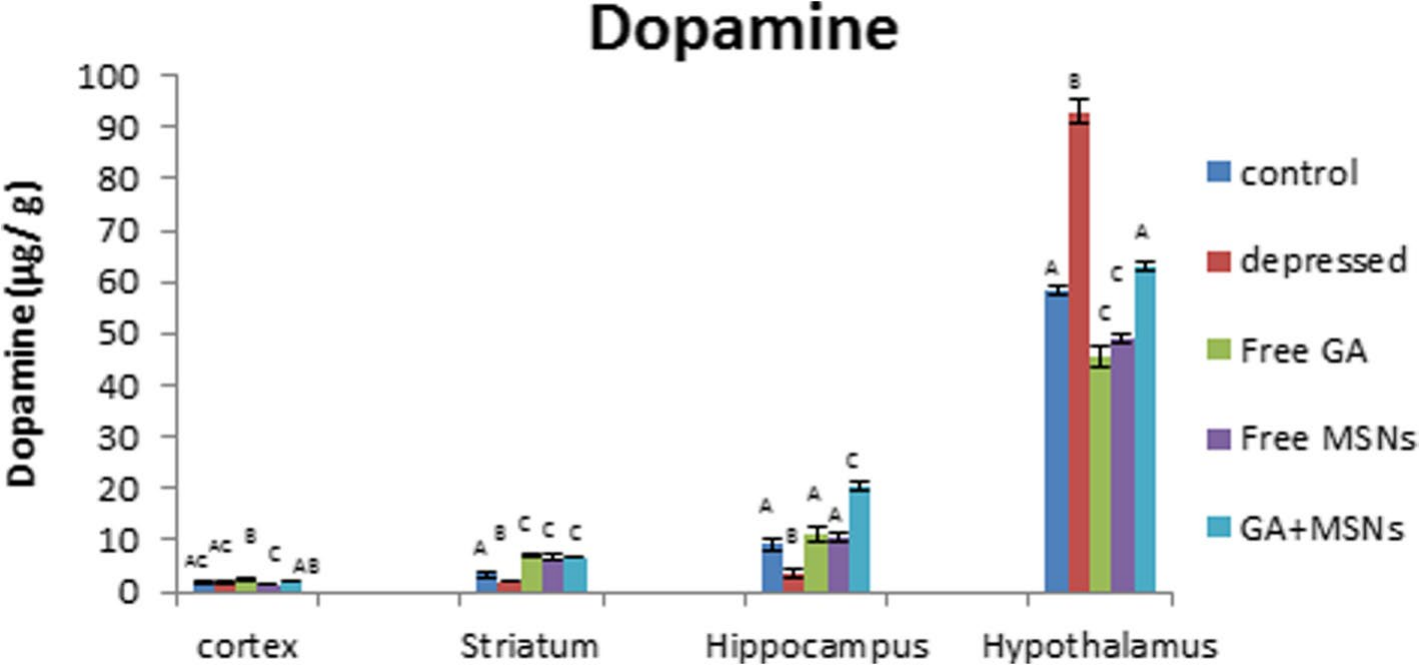
Effect of i.p. injection of reserpine, free GA, free MSNps, GA-loaded MSNPs on the DA levels in different brain areas of adult male Wistar rats

In the reserpine-induced rat depression model, a significant reduction in DA levels was detected in the hippocampus and striatum (− 59 and − 40%, respectively) compared with normal levels (Fig. 11), whereas a significantly significant increase in DA was detected in the hypothalamus. Treatment with free GA significantly increased levels of DA in the cortex (36%) and the striatum (117%) and insignificantly in the hippocampus compared with normal values. Treatment with free GA failed to restore DA levels to that of the controls in the hypothalamus. Comparison between the depressed untreated and free GA-treated groups revealed a significant increase in DA levels in the cortex and hippocampus, whereas a significant reduction in the hypothalamus was observed. DA levels were elevated significantly in the hippocampus and striatum and when the rats from the depressed group were treated with free MSNs (15 and 101%, respectively) compared with the controls. However, free MSNs failed to restore DA levels to that of the controls in the hypothalamus.

Compared with untreated depressed rats, free MSNs failed to increase DA levels in the cortex and hypothalamus but increased hippocampal and striatal DA levels. Treatment with GA-loaded MSNs restored DA levels in the cortex and hypothalamus to that of the controls. In the hippocampus and striatum, GA-loaded MSN treatment increased DA levels significantly (123 and 100%, respectively) compared with control values. Compared with the depressed untreated rats, GA-loaded MSN treatment increased DA levels significantly in the hippocampus and striatum, insignificantly in the cortex, and significantly reduced DA levels in the hypothal reduction in DA levels were observed in the hippocampus.

#### Oxidative stress parameters

**Fig. 12:**
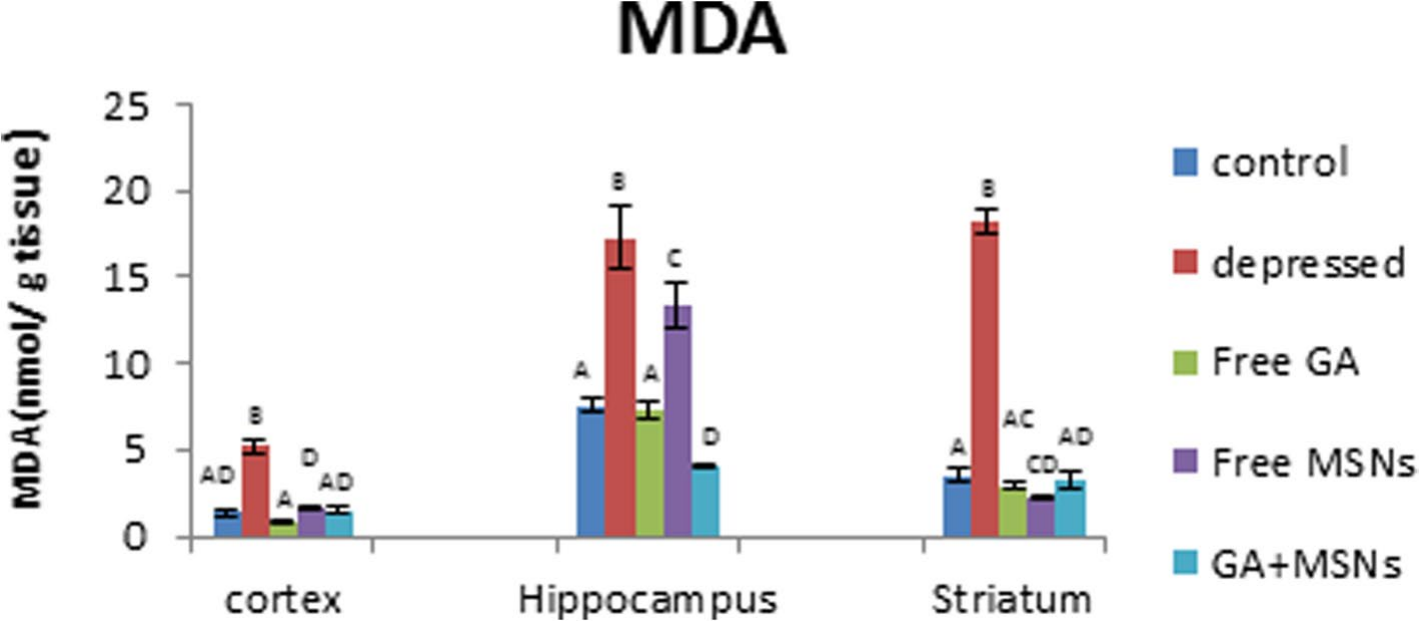
Effect of i.p. injection of reserpine, free GA, free MSNps, GA-loaded MSNPs on the malondialdehyde (MDA) levels in different brain areas of adult male Wistar rats

As shown in Fig. 12, a significant increase in MDA values was evident in the cortex (287%), hippocampus (127%), and striatum (415%) regions in the reserpine-induced rats compared with the controls. Compared with the depressed untreated rats, treatment using free GA, free MSNs, and GA-loaded MSNs resulted in significantly reduced MDA values in the cortex, hippocampus, and striatum. Free GA treatment decreased MDA levels to that of the controls in these three regions. Free MSN treatment for depressed rats restored cortical MDA levels and significantly decreased striatal MDA levels by 39% but did not restore hippocampal MDA levels to that of the controls. When GA-loaded MSNs were used to treat depressed rats, MDA levels were restored to normal-like levels in the cortex and striatum. Furthermore, the hippocampal MDA levels decreased significantly (47%) compared with the control rats.

**Fig. 13:**
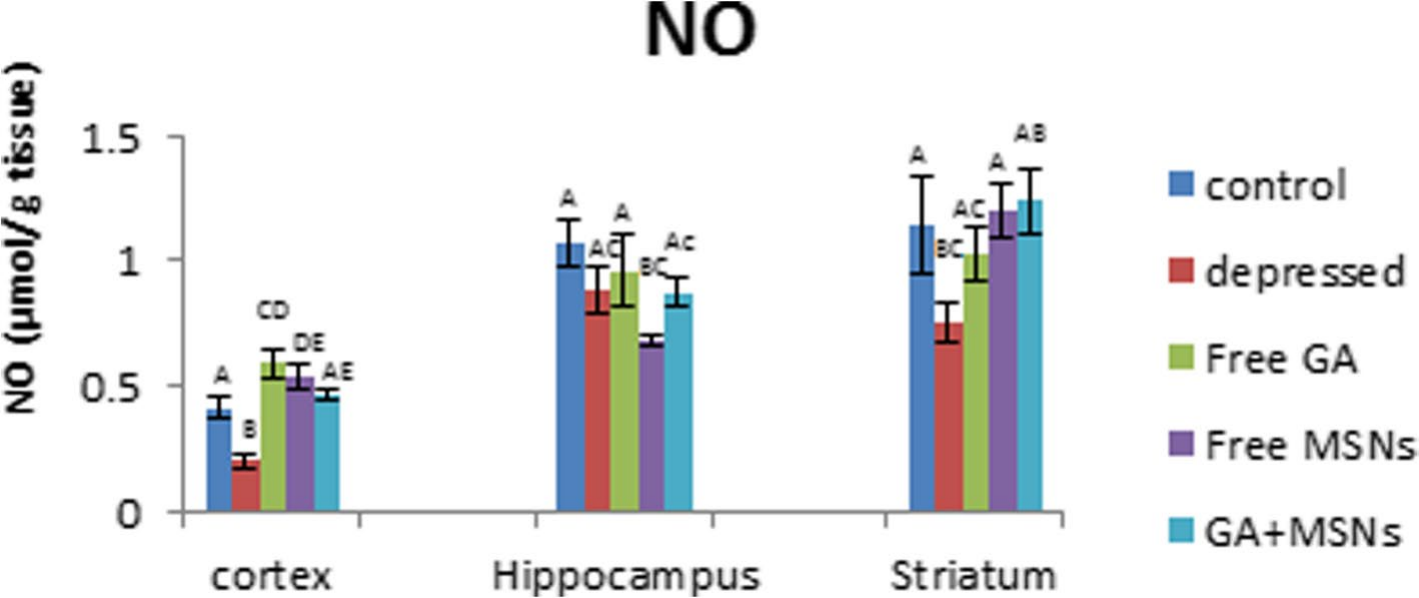
Effect of i.p. injection of reserpine, free GA, free MSNps, GA-loaded MSNPs on the nitric oxide (NO) levels in different brain areas of adult male Wistar rats

Figure 13 shows a significant reduction in NO values in the cortex and striatum in depressed rats (− 51 and − 34%, respectively). Treatment with free GA restored striatal NO levels to control-like values and increased cortical NO significantly (44%) more than the control. In the cortex and striatum, free MSNs reversed the decreased NO levels induced by reserpine to significantly elevated levels of 32 and 5%, respectively, and significantly decreased hippocampal NO levels compared with normal values. GA-loaded MSN treatment resulted in an insignificant change in NO levels in the cortex, striatum, and hippocampus compared with normal controls.

**Fig. 14:**
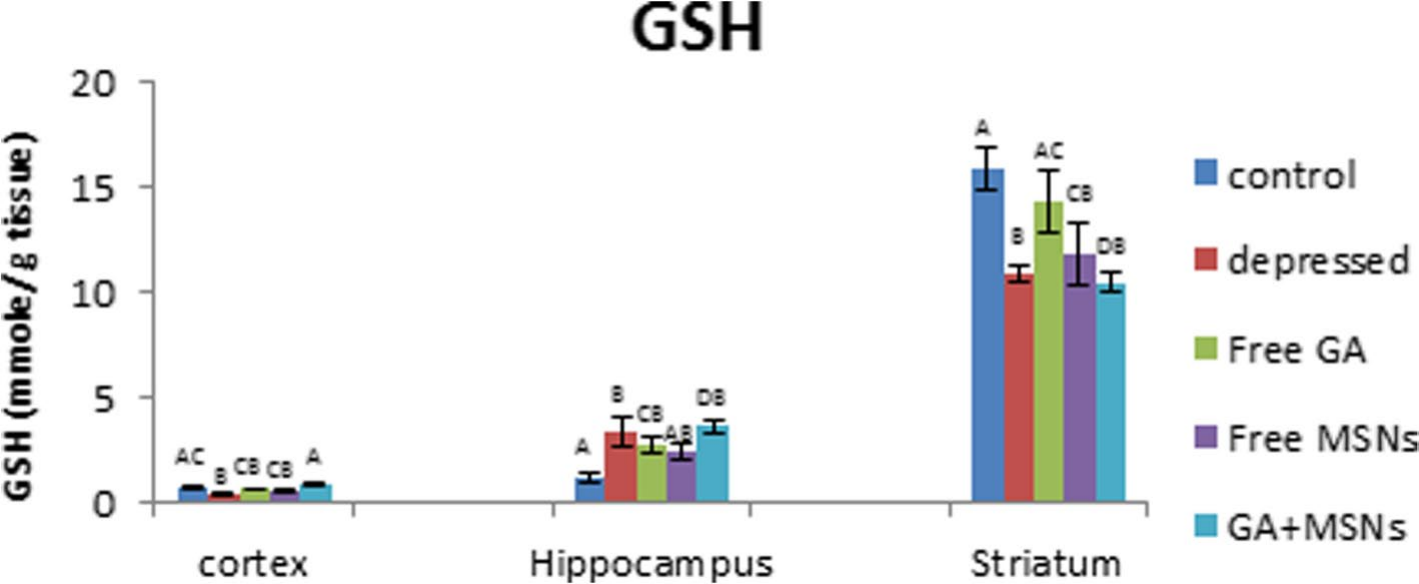
Effect of i.p. injection of reserpine, free GA, free MSNps, GA-loaded MSNPs on the reduced glutathione (GSH) levels in different brain areas of adult male Wistar rats

A significant reduction in GSH values in the cortex (− 37%) and striatum (− 31%) and a significant elevation in hippocampal GSH values (188%) were detected in the depressed rat compared with the controls (Fig. 14). Treatment with free GA restored GSH values in the cortex and striatum to control levels and maintained the increased hippocampal GSH levels. Treatment of depressed rats with free MSNs resulted in insignificant changes in GSH levels in the cortex and hippocampus and a significant reduction in the values of striatal GSH (− 26%) compared with controls. GA-loaded MSN treatment increased GSH values significantly in the hippocampus (213%) and insignificantly in the cortex, whereas significantly reduced GSH levels were detected in the striatum compared to the control group.

**Fig. 15:**
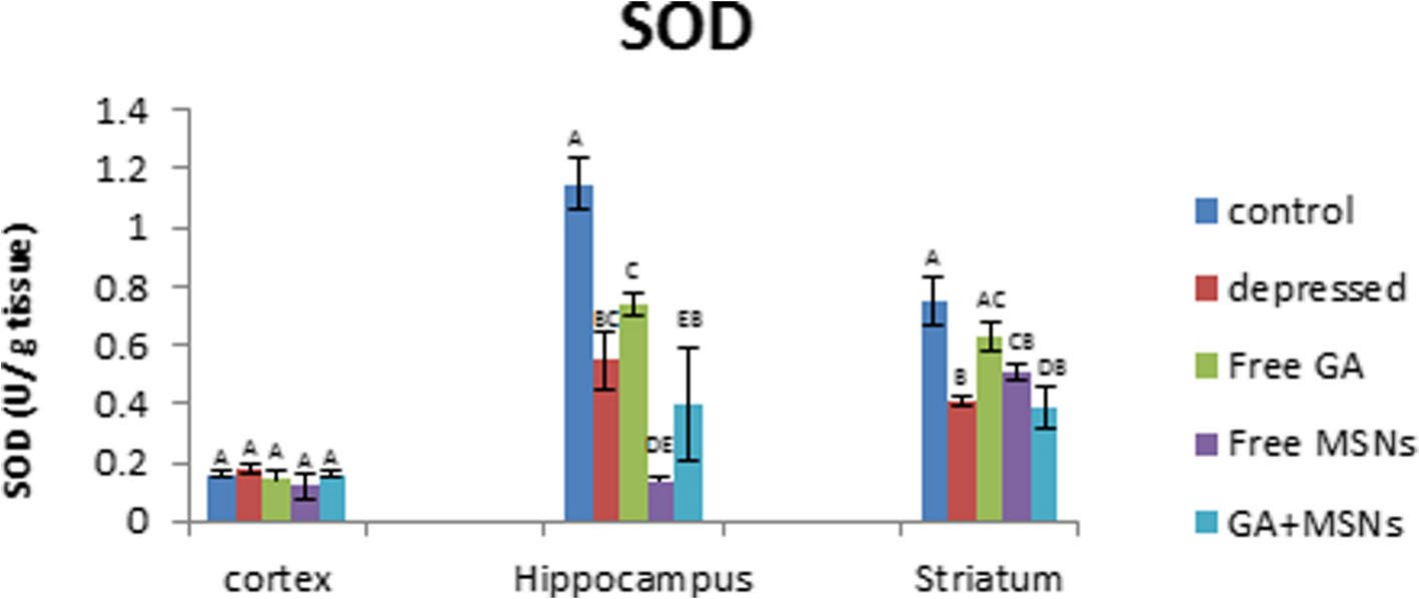
Effect of i.p. injection of reserpine, free GA, free MSNps, GA-loaded MSNPs on the superoxide dismutase (SOD) levels in different brain areas of adult male Wistar rats

Reserpine injection significantly decreased SOD activity in the hippocampus and striatum area by 52 and 45%, respectively, compared with control values (Fig. 15). Treatment with free GA restored SOD activity in the cortex and striatum regions to normal values and did not affect decreased hippocampal SOD activity induced by reserpine. Treatment with free MSNs decreased the activity of SOD insignificantly in the cortex and significantly in the hippocampus and striatum (− 88 and − 32%, respectively) compared with control rats. GA-loaded MSN treatment restored cortical SOD activity to control values and induced a significant reduction in the activity of SOD in the hippocampus and striatum by 65 and 48%, respectively, compared with the controls. Compared with the depressed untreated group, treatment by using free GA, free MSNs, and GA-loaded MSNs showed insignificant alterations in the activity of SOD in the cortex and hippocampus. The activity of SOD raised significantly with free GA and insignificantly with free MSN in the striatum and decreased insignificantly following treatment with GA-loaded MSNs.

**Fig. 16:**
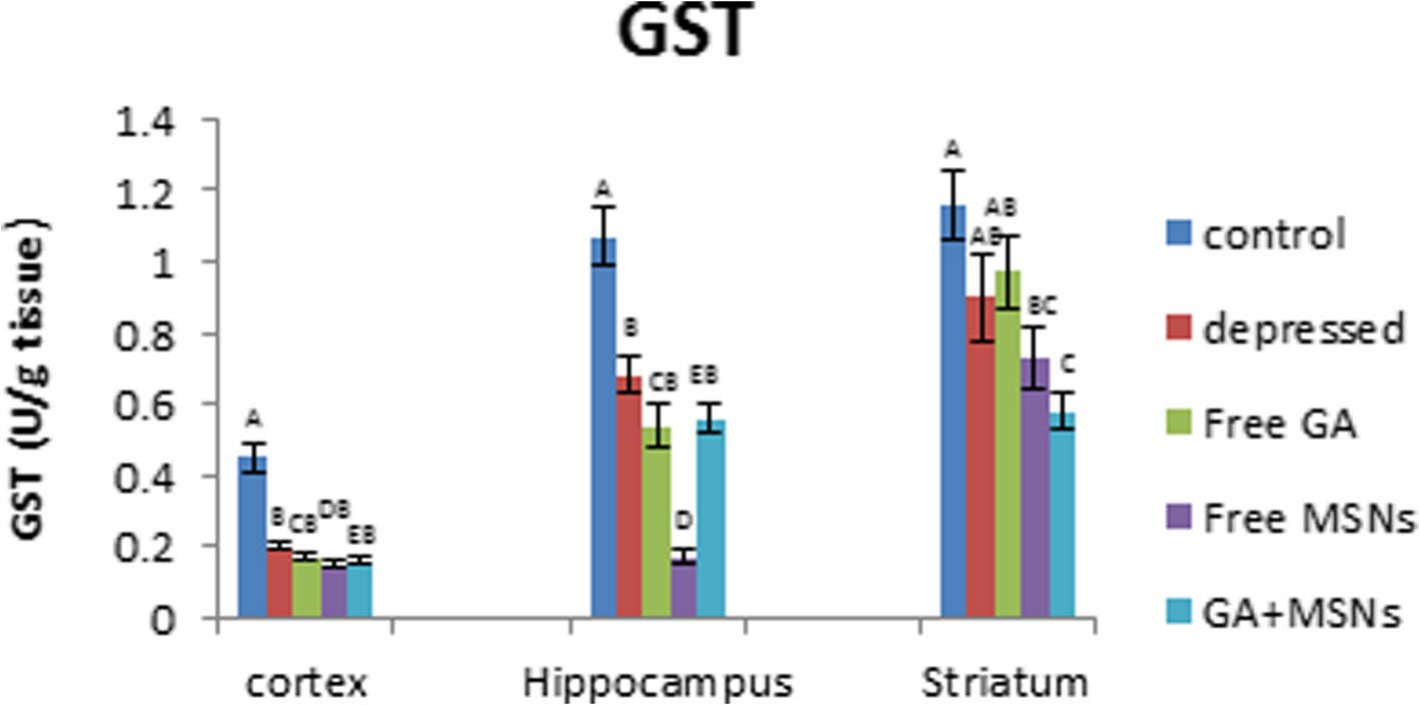
Effect of i.p. injection of reserpine, free GA, free MSNps, GA-loaded MSNPs on the glutathione-s-transferase (GST) levels in different brain areas of adult male Wistar rats

A significant reduction in the activity of GST was detected in the cortex (− 56%) and hippocampus (− 36%), whereas an insignificant decrease was found in the striatum of the depressed rats in comparison to the controls (Fig. 16). Compared with untreated depressed rats, GST activity exhibited an insignificant change in the cortex, hippocampus, and striatum in rats treated using free GA, free MSNs, and GA-loaded MSNs. However, a significant reduction in GST activity in the hippocampal and striatal areas was observed. Free GA, free MSNs, and GA-loaded MSN treatment did not successfully restore reduced GST activity caused by reserpine treatment in the three brain regions.

**Fig. 17:**
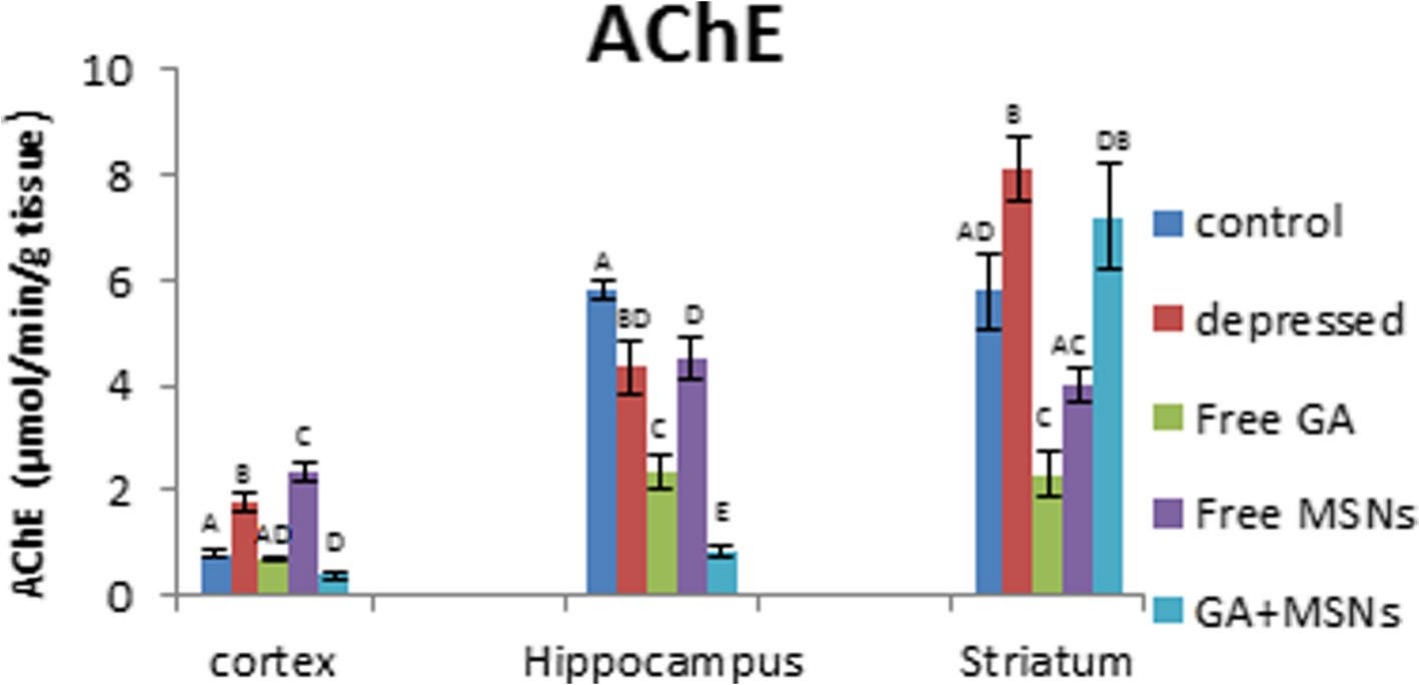
Effect of i.p. injection of reserpine, free GA, free MSNps, GA-loaded MSNPs on the Acetylcholine esterase (AChE) levels in different brain areas of adult male Wistar rats

A significant elevation in the activity of AChE in the cortex and striatum was observed in the depressed rats (121 and 40%, respectively), whereas the activity of AChE decreased in a significant manner (25%) in the hippocampus compared with the controls (Fig. 17). Free GA treatment restored AChE activity in the cortex, whereas significantly decreased enzyme activity was observed in the hippocampus and striatum, respectively (59 and 60%), compared to the controls. Treatment with free MSN caused a significant elevation in the activity of AChE in the cortex (194%), a significant reduction in the hippocampus area (− 22%), and an insignificant reduction in the striatum compared with normal values. GA-loaded MSNs reduced AChE activity in the cortex and hippocampus by 50 and 86%, respectively, and induced an insignificant increase in the striatal enzyme activity compared with controls. Compared with untreated depressed rats, treatment with free GA or GA-loaded MSNs resulted in a significant reduction in the activity of AChE in the cortex, hippocampus, and striatum. In addition, the free MSN-treated group exhibited a significant rise in AChE activity in the cortex and insignificant changes in the hippocampus and striatum.

## Discussion

To overcome this challenge in drug delivery, scientists have used nanotechnology as a treatment strategy. The BBB (blood-brain barrier) is the most potent membrane in the body. It regulates exogenous substances’ transport and prevents drugs from crossing the barrier into the brain [45]. The entry mechanism by silica nanoparticles is influenced by physicochemical factors, including zeta potential and particle size [46]. In previous studies, MSNs have been shown to penetrate the BBB and get to the brain via endocytosis or other carriers [47, 48]. Therefore, the form, charge, and size of MSNs seem to be the most important factors to consider when evaluating their design and efficacy as delivery vehicles for brain medication.

The biomedical uses of MSNs are affected by interactions between nanoparticles and cells in tissues and lipid bilayers. In addition, the interactions between MSNs and media constituents may theoretically affect their influence on biological tissues and cells. The surface properties of MSNs almost entirely determine such interactions. MSNs, as similar to other nanomaterials, tend to agglomerate or aggregate during it is distributed in any liquid medium such as water [49, 50]. Therefore, characterizing the surface characteristics of MSNs in both the dry and wet states is essential [51].

The index of polydispersion (PDI) measures particle size uniformity and homogeneity. A narrow size dispersion is represented by PDI levels ranging from 0.1 to 0.5, whereas PDI values above 0.5 refer to a wide dispersion [52]. Such systems used for medication require a low value of PDI to improve pharmacokinetic characteristics, including absorption and dispersion [53]. These results showed that the polydispersity index (PDI) values for MSNs and GA-loaded MSNs were 0.447 and 0.48, respectively, suggesting that free MSNs and GA-loaded MSNs have a perfect uniform distribution of particle size.

The potential is crucial for determining agglomeration against colloidal stability [54].The potential zeta is a fundamental measure of a colloidal dispersion’s stability. The zeta potential scale measures how far repulsive forces between neighboring, identically charged particles disperse. A high zeta potential imparts tiny molecules, and the solution or dispersion can avoid particles with more excellent stability and aggregation. The minimal potential enables the forces of attraction to surpass this repulsiveness. Also, the dispersion may be broken and flocculated. As a result, a zeta potential that is high colloid (negative or positive) is stabilized electrically, whereas a zeta potential that is low colloid can flocculate or coagulate [55, 56]. In this study of MSNs and GA-loaded MSNs, zeta potentials were negative (− 29.9 and − 31.4 mV, respectively). These results indicate stable dispersions of MSNs with negatively charged surfaces in water.

In addition to zeta potential, non-aqueous colloid stability is also influenced by particle size, and the most significant silica samples appear to be the most stable [57]. Hydrodynamic diameters are always more remarkable than the particle size under dry conditions [58]. According to our data, the rate of release of GA from MSNs was fast throughout the first 24 hours. Then declined in the final 48 hours. The original quick release of GA may be attributed to its discharge from the surface of the nanoproduct, and its postponed release may occur through chemical bond hydrolysis. GA was chemically attached to silica nanostructures, and a therapeutic delivery method based on silica nanoparticles was created. GA may be progressively released from the GA-MSNs according to in vitro release studies [31].

This study showed the creation of a reserpine-induced animal study of depression. The reduction in the period of immobility may corroborate this model. In this study, three therapies (free GA, free MSNs, and GA-loaded MSNs) were tested for antidepressant activity using this model.

GA is a common polyphenol in crops, berries, mangos, walnuts, green tea, and wine [13]. In animal research, GA showed anti-anxiety properties [14]. GA is soluble in alcohol and ether but has low solubility in water [59]. It is characterized by short-lived and aqueous GA auto-oxidation, which considerably lowers residence duration and bioavailability in humans. Thus, we enhanced GA bioavailability in the present study by loading it into MSNs to control its release through hydrolysis of the chemical bonds between GA and MSNs [31]. One possible mechanism through which GA acts is gamma-aminobutyric acid transaminase (GABA-T) activity, which inhibits inducible nitric oxide synthase (NOS) and non-neuronal NOS involved in nitrergic regulation. Increased neurotransmission mediated by GABA has been linked to reducing anxiety, as described in the study by Gilhotra and Dhingra [60].

FST and OFT were used to evaluate the behavioral effects of antidepressant activity. By assessing reduced immobility times in FST, one can predict antidepressant potential, and the number of squares that have been crossed, rearing, and grooming in OFT may be used to evaluate antidepressant drugs. In this study, rats injected with reserpine exhibited a more significant period of immobility in the FST, and the number of squares that were crossed and rearing and grooming were reduced compared with control rats. As a result, depression symptoms were present. Treatment with free GA, free MSNs, and GA-loaded MSNs (20 mg per kg, i.p.) for 10 days resulted in a slight reduction in immobility period in the FST compared with the depressed untreated rat. However, in the OF test, the same treatment resulted in noticeable differences in rearing and grooming compared with the normal rats, whereas only treatment with free GA showed an insignificant increase in the crossed squares number compared with depressed untreated rats. This indicates a slight improvement in the behavior of depressed rats appearing after this short period of treatment. The complete recovery of the behavioral changes induced by reserpine may require prolonged treatment with these agents.

Lipid peroxidation was a crucial factor in the establishment of toxicity and a manifestation of damage caused by oxidation [61]. Pharmacological modulation of the NO pathway may lead to novel therapeutic options for various neuropsychiatric diseases [62]. On the other hand, NO has been shown to have dual functions in biological systems. It acts as an oxidant and an antioxidant [63, 64]. In the current study, MDA levels were insignificantly lower in the cortex, striatum, and hippocampus of rats injected with free GA.

Furthermore, NO levels in these three regions were reduced below normal. Proxy nitrite production is reduced when NO concentrations are reduced, which reduces tissue peroxidation and quenches MDA formation [65]. Loading GA into MSNs reduced MDA levels significantly in the hippocampus to a lesser extent than GA in the free form. This may enhance the activity of antioxidants which relieve the oxidative stress that has been created. MDA levels in the hippocampus and striatum decreased concomitantly with increasing NO levels. Kanner et al. [66] proposed that increasing NO levels may prevent oxidative damage by serving as an electron donor, reducing free radical generation, and lowering MDA to normal values. Reduced glutathione is an essential cellular antioxidant protection agent that prevents oxidative damage produced by reactive oxygen species (ROS) in the brain [67].

GSH is an antioxidant that detoxifies exogenous and endogenous harmful chemicals by altering their solubility. In non-enzymatic reactions, GSH interacts with free radicals directly and functions as a source of electrons to reduce peroxides produced by glutathione peroxidase. Glutathione disulfide (GSSG) is generated due to oxidation, and glutathione reductase converts it to GSH [68]. Glutathione S-transferases also utilize GSH to detoxify electrophilic substances, providing cells with multiple defensive functions versus ROS and its metabolites [69]. For the GSH-mediated defense mechanism, GST is an enzyme that exploits the sulfur atom of GSH to interact with hazardous chemicals [70]. We observed that GSH levels were reduced by free GA treatment in the cortex, striatum, and hippocampus. Thus a decrease in GSH values may be associated with its use in defending cells against harmful free radicals [71]. GST activity in the cortex, hippocampus, and striatum was decreased by free GA. This may be related to its activity in reducing oxidative stress [72]. Loading GA into MSNs resulted in GSH values in the cortex and hippocampus, whereas a significant decrease in GST values in both tissues was observed. GST activity may decrease significantly due to its inability to sustain the conversion of GSH to an oxidized state to withstand oxidative stress [73, 74].

Superoxide anion (O2) is converted to hydrogen peroxide (H2O2) by SOD, which is subsequently transformed to molecular oxygen and water by glutathione peroxidase or catalase [75]. Treatment with free GA or GA-loaded MSNs caused a reduction in the SOD activity in this study. Thus, a decrease in the activity of SOD following treatment using free GA and GA-loaded MSNs may be attributed to its consumption during O_2_ scavenging, in which it is subsequently converted into H_2_O_2_ by catalase or peroxidase enzymes.

Acetylcholinesterase (AChE) inhibits synapses’ chemical transmission by hydrolyzing acetylcholine neurotransmitters [76]. In the current study, the AChE activity was reduced significantly in the hippocampus area of depressed rats compared with the control group. Decreased AChE activity is associated with a risk of developing depression [77]. According to Tsakiris et al. (2000b), AChE is extremely sensitive to free radicals, which inhibit enzyme activity [78]. This may explain the observed decrease in the activity of AChE. However, the significantly lowered AchE activity occurred in the hippocampus area, but reserpine caused significant increases in AChE activity in the cortex and striatum in the depression rat model. Administration of reserpine caused a significant increase in ACh content, which may increase cholinergic activity [79]. GA enhanced AChE activity in the cortex. According to earlier research, nanostructures may bind to acetylcholinesterase and modify their activity once entering the body, and AchE is an essential enzyme in mammalian neurological functions [80, 81]. This may cause increased AchE activity following treatment with free GA or GA-loaded MSNs. While free GA reduced AChE activity in the hippocampus and striatum, GA-loaded MSNs lowered the activity of AChE in the cortex and hippocampus. The decrease observed in the activity of AChE in the current study could be associated with its exhaustion in mitigating increased cholinergic activity. AChE appears to be responsible for terminating ACh’s action at its receptors through hydrolysis [82].

Furthermore, GA-loaded MSNs significantly increased AChE activity in the striatum. High AChE activity promotes choline uptake by hydrolyzing ACH [83]. One of the most cholinergic regions in the brain is the striatum which contains high concentrations of all cholinergic markers [84, 85]. The striatum exhibited the highest AChE activity because of the high levels of acetylcholine and AChE released spontaneously from cholinergic interneurons in this part of the brain [86].

Monoamines, such as dopamine, serotonin, and norepinephrine, regulate brain functions in animals and humans [87]. In this study, chronic reserpine exposure resulted in a decrease in monoamine levels. Reserpine reduces monoamines by irreversibly inhibiting their vesicular monoamine carrier and inhibiting monoamine from being repackaged into vesicles after being liberated from the synapses [88]. The metabolizing enzyme monoamine oxidase can break down monoamine neurotransmitters [89]. In the central nervous system, the metabolic degradation of serotonin, catecholamine, and other endogenous amines is carried out by monoamine oxidase. During the depression, the MAO levels in the brain increase, which results in lower monoamine levels [90].

Furthermore, in the cortex region, free GA and GA-loaded MSNs restored serotonin to a control-like level in this study. Only GA-loaded MSNs could increase the reduced value of NEP. The other treatments resulted in increased DA levels above the normal level. The enzyme monoamine oxidase is inhibited by GA [15]. Similarly, all three treatments increased DA levels in the hippocampus compared to the control. In the striatum, lower values of serotonin and dopamine in the GA-treated group were increased above the control value, and the NEP was raised to a level similar to that of the control.

GA was provided in a formulation (GA-loaded MSNs) that was more water-soluble to enhance these effects and enhance GA bioavailability. Furthermore, GA-loaded MSNs significantly increased striatal monoamine levels, whereas free MSNs only increased DA levels. GA-loaded MSNs increased serotonin, norepinephrine, and dopamine levels in the hypothalamus, whereas free GA raised only serotonin levels to that of the control, whereas free MSNs raised NEP levels only. As a result, the increase in serotonin was observed with GA and the GA-loaded MSN formulation. In the current study, it is possible that link to a significant GA-induced decrease in MAO-A activity [91]. This enzyme degrades monoamine neurotransmitters and prefers serotonin as a target, so antidepressant monoamine oxidase inhibitor drugs target MAO-A [92]. The formulation also has a more significant effect in some brain areas because loading GA into MSNs alters its delivery properties. These data suggest that GA-loaded MSNs are more successful than free GA and free MSNs in restoring reserpine-induced monoamine deficiency in the four examined brain regions.

## Conclusion

MSNs were introduced as effective nanoplatforms for delivering medications to the brain. Various physical methods were used to characterize the formulation, and its characteristics were determined to be appropriate for drug carriers. The MSNs created in this study were capable of crossing the BBB. The results highlight the promising antidepressant effects of GA in the current reserpine-induced depression model in rats by reducing oxidative stress and elevating monoamine levels. This study also demonstrated that loading GA on MSN nanoparticles enhances its antidepressant effects. The findings of this study may pave the way for MSNs to be used as a feasible approach for directing medicines to specific brain regions for a range of diagnostic and therapeutic purposes. However, more research is needed to establish GA-loaded MSNs as one of the main treatments for depression.

## Data Availability

All data needed to support the conclusions are included in this article, and supplementary data are present in the supplemental materials. Additional data related to this paper can be requested from the author (hfahmy@sci.cu.edu.eg).

## Abbreviations

NO: Nitric oxide
5-HT: Serotonin (5-hydroxytryptamine)
Acetyl-CoA: Acetyl coenzyme A
ACh: Acetylcholine
AChE: Acetylcholinesterase
AGE: Advanced glycation end-products
ALE: Advanced lipoxidation end-products
BBB: Blood brain barrier
BCEC: Brain capillary endothelial cells
CAT: Catalase
CDNB: 1-choloro-2,4-dinitrobenzene
cp: centipoise
CTAB: N- cetyl tri methyl ammonium bromide
DA: Dopamine
DHBA: Di hydroxybenzoic acid
DLS: Dynamic light scattering
DMSO: Dimethyl sulfoxide
DNA: Deoxyribonucleic acid
DTNB: Dithiobisnitrobenzoic acid
FST: Forced swimming test
GA: Gallic acid
GABA-T: Gamma aminobutyric acid transaminase
GSH: Reduced glutathione
GSH-PX: Glutathione peroxidase
GSSG: Oxidized Glutathione
GSTs: Glutathion-s-transferase
HPA: Hypothalamus – pituitary – adrenal glands
IFN-γ: Interferon-gamma
INT: 2-(4-idophenyl)-3-(4-nitrophenol)-5-phenyltetrazolium
IP: Intraperitoneal
LD: Lethal dose
LPS: Lipopolysaccharide
MAO-A: Monoamine oxidase TYPE A
MAO-B: Monoamine oxidase TYPE B
MAOIs: Monoamine oxidase inhibitors
MDA: Malondialdehyde
MDD: Major depressive disease
MSNs: Mesoporous silica nanoparticles
mV: Milli volts
NA: Noradrenaline
NADPH: Nicotinamide adenine dinucleotide phosphate
NaOH: Sodium hydroxide
NaSSAs: Noradrenergic and specific serotonergic antidepressants
NE: Norepinephrine
NED: Naphthyl ethylene diamine di hydrochloride
NET: Norepinephrine transporter
Nm: Nanometer
NOS2: Nitric oxide synthase 2
NPs: Nanoparticles
NRIs: Noradrenaline reuptake inhibitors
O2• −: Superoxide
OFT: Open field test
PBS: Phosphate-buffered saline
PDI: Polydispersity index
PMS: Phenazine methosulfate
RIMAs: Reversible monoamine oxidase
RNS: Reactive nitrogen species
ROS: Reactive oxygen species
Rpm: Round per minute
S.E.M: Standard error of the mean
SAD: Seasonal affective disorder
SEM: Scanning electron microscopy
SERT: Serotonin transporter
SNRIs: Serotonin and noradrenaline reuptake inhibitors
SOD: Superoxide dismutase
SSRIs: Selective serotonin reuptake inhibitors
TBA: Thiobarbituric acid
TBARS: Thiobarbituric acid reactive substances
TCAs: Tricyclic antidepressants
TeCAs: Tetracyclic antidepressants
TEOS: Tetra ethoxy ortho silicate
TNB: 2-nitro-5-thiobenzoic acid
